# A new pathway for elective surgery to reduce cancellation rates

**DOI:** 10.1186/1472-6963-12-154

**Published:** 2012-06-11

**Authors:** Einar Hovlid, Oddbjørn Bukve, Kjell Haug, Aslak Bjarne Aslaksen, Christian von Plessen

**Affiliations:** 1 Institute of Social Science, Sogn og Fjordane University College, Postbox 133, 6851, Sogndal, Norway; 2 Department of Public Health and Primary Health Care, University of Bergen, Bergen, Norway; 3 Department of Radiology, Haukeland University Hospital, Bergen, Norway; 4 Institute of Surgical sciences, University of Bergen, Bergen, Norway; 5 Department of Thoracic Medicine & Infectious Disease, Hillerød Hospital, Hillerød, Denmark; 6 Institute of Medicine, Faculty of Medicine and Dentistry, University of Bergen, Bergen, Norway; 7 Department of Health Studies, Faculty of Social Sciences, University of Stavanger, Stavanger, Norway

**Keywords:** Quality improvement, Process redesign, Cancellation of surgery, and Health information technology

## Abstract

**Background:**

The cancellation of planned surgeries causes prolonged wait times, harm to patients, and is a waste of scarce resources. To reduce high cancellation rates in a Norwegian general hospital, the pathway for elective surgery was redesigned. The changes included earlier clinical assessment of patients, better planning and documentation systems, and increased involvement of patients in the scheduling of surgeries. This study evaluated the outcomes of this new pathway for elective surgery and explored which factors affected the outcomes.

**Methods:**

We collected the number of planned operations, performed operations, and cancellations per month from the hospital’s patient administrative system. We then used Student's *t-*test to analyze differences in cancellation rates (CRs) before and after interventions and a u-chart to analyze whether the improvements were sustained. We also conducted semi-structured interviews with employees of the hospital to explore the changes in the surgical pathway and the factors that facilitated these changes.

**Results:**

The mean CR was reduced from 8.5% to 4.9% (95% CI for mean reduction 2.6-4.5, *p* < 0.001). The reduction in the CR was sustained over a period of 26 months after the interventions. The median number of operations performed per month increased by 17% (*p* = 0.04). A clear improvement strategy, involvement of frontline clinicians, introduction of an electronic scheduling system, and engagement of middle managers were important factors for the success of the interventions.

**Conclusion:**

The redesign of the old clinical pathway contributed to a sustained reduction in cancellations and an increased number of performed operations.

## Background

Cancellation of planned surgeries is a known quality problem in healthcare that harms patients and wastes resources, leading to increased healthcare costs [1,2]. Cancellation rates (CRs) vary in different settings, from less than 1% to as high as 23% [1,3,4]. It has been suggested that more than half of cancellations can be avoided [4,5].

Reasons for cancellations are complex because they are related to patients, organizational issues, and clinical staff. The main reasons are patient no-shows, patients’ medical conditions, overbooking of lists, and facility shortcomings [1,2,4]. Redesigning work processes, improving management, and performing early clinical evaluation of patients have been suggested to reduce CRs [6]. Various studies have shown that improved and early preoperative assessment prior to surgery reduces CRs [7-10]. These studies have demonstrated a reduction in CRs that was primarily attributable to earlier and improved preoperative assessment. Nonetheless, a paucity of information exists regarding the long-term effects of combined interventions that include elements other than just improved medical preoperative assessment.

We studied a Norwegian district general hospital in a rural community of 10,000 inhabitants. The hospital has 7 operating suites, 34 surgical beds, and serves a population of 107,000. Within the area, there are also two smaller district hospitals. Healthcare in Norway is financed by the state and most hospitals are publicly owned. They are geographically organized as regional and local health authorities. Before the changes were made to the pathway for elective surgery, CRs were high and fluctuating, resources were not optimally used, patient information was unclear, and patients complained about the duration of time spent waiting for surgery outside the hospital.

In 2007, the board of the regional health authority decided to increase the number of day surgeries. Middle managers in the hospital used this decision as leverage to start an improvement project to redesign the entire pathway for elective surgery. At the same time, the top management of the local health authority decided to work more systematically with quality improvement in general, and developed a common strategy for conducting quality improvement projects. The strategy was not based on any particular theoretical model, although it was influenced by the Model of Improvement [11]. The aim was to involve frontline professionals in the detection of systemic problems and to improve clinical processes. All improvement projects were meant to address professional, patient, and management quality (resource utilization), as well as staff satisfaction [12]. A small administrative unit was established to support frontline professionals in running the improvement projects.

Often the follow-up times of studies on quality improvement are too short to demonstrate sustainability of changes [13]. Therefore, we used data over a five-year period. We describe how multifaceted interventions across different departments led to a sustained reduction of cancelled operations. Furthermore, we explore contextual factors and their importance for sustaining these improvements.

## Methods

### Planning of interventions

The project involved all of the surgical departments at Førde Hospital, including in- and outpatient ophthalmology, general surgery, gynecology, orthopedics, and ear, nose, and throat. Additionally, the hospital has a small odontology unit that accounted for less than 5% of the total operations.

Four different project groups were established to improve different aspects of the elective surgery pathway. Redesign of this pathway was the first project that was run in accordance with the new improvement strategy of the local health authority. Table 1 displays the main activities during the project period. A strong motivator for the leaders who initiated the project was to improve patient satisfaction. Patient advocacy groups were invited to take part in the project groups but declined to participate. Instead, actual patient cases were used to focus improvement efforts, and patient-centered interventions were a core idea in the redesign process [14,15].

**Table 1:** Main events during the project period

**Time**	**Activity**
June 2007	Decision by the board of the local health authority to increase the number of day surgeries
September 2007	Report from work group suggests measures to increase day surgeries in a new day-surgery center
Fall 2007	Decision by top management of the local health authority to work more systematically with quality improvement and develop a common strategy for conducting quality improvement projects
	Establishment of a small unit to support frontline professionals in running improvement projects
January 2008	Remodeling of premises for a day-surgery center
February 2008	Project groups redesign the pathway for elective surgery
March 3, 2008	Opening of day-surgery center and implementation of new clinical pathway
April 2008	Implementation of electronic system for scheduling and planning surgery
2008-2010	Follow-up and adaptation of interventions to sustain improvement

The entire pathway for elective surgery was redesigned, focusing on earlier patient assessment, improved communication between staff, improved management, improved planning, and patient participation in the planning of their elective operations. Table 2 shows a description of the clinical pathway and a detailed description of the different intervention elements, including the intended improvements. Ideas for improvements stemmed from discussions in the project groups, recommendations in the literature, and a site visit to a hospital with low cancellation rates.

**Table 2:** Main steps of the pathway for elective surgery before and after redesign

**Time period**	**Clinical pathway before intervention**	**Intervention**	**Intended improvement**
Before consultation at outpatient clinic	Referrals for elective surgery were sent to various departments. Each surgical department had their own lists of patients who were waiting for a consultation and surgery.	One electronic reception for all referrals for elective surgery.	Waiting list transparent across departments. More unified handling of referrals.
Consultation at outpatient clinic	Patients cleared for surgery were sent home without an appointment for surgery and without a medical pre-assessment.	New routine that clarified the allocation of work between surgical and anesthesia personnel with regard to clinical pre-assessment of the patient.	Earlier and improved medical pre-assessment is known to reduce cancellations.
			Patient participation in planning date for surgery may improve patient satisfaction. Early notice of date for surgery is suggested in the literature as a factor that might reduce no-shows.
	Medical pre-assessment was done the day before surgery.		
		Patients participate in planning the date of surgery and obtain the actual appointment while at the outpatient clinic.	
Consultation at drop-in anesthesia outpatient clinic at day-surgery center	Not applicable	A new day-surgery center is created within the existing premises.	Improved information flow between surgical and anesthesia personnel may improve the quality of the clinical process.
		Patients cleared for surgery proceed straight to the laboratory for blood sampling and medical pre-assessment at newly established drop-in anesthesia outpatient clinic at the day-surgery center.	
		The surgeon’s considerations are written immediately after the consultation so that anesthesia personnel have the preoperative information during the preoperative assessment.	
Preparing for surgery	Letter to patient with appointment for surgery. Patient had no influence on appointment time.	Patient receives phone call from hospital 2 days prior to surgery to ensure that he is fit and ready.	Patients get a reminder of their appointment, which can reduce cancellations due to no-shows. If the patient is temporarily ill, then there is time to call a new patient and avoid a cancellation.
	Limited planning between different surgical departments. Each surgical department had their own surgery program that basically was a text file.	One common electronic surgery planning system for all departments. Designated coordinator supervises the planning process between departments.	
			One common overview for all departments allows better coordination and planning and might lead to more operations per day. Cancellations caused by facility shortcomings, such as double-booking of the same equipment, may be reduced.
Surgery	Patient showed up for pre-assessment the same day or one day in advance of the planned surgery. Routines varied between departments.	All patients scheduled for elective surgery are received at the day-surgery center. New standardized routines are implemented for pre-surgery preparations.	Centralizing all surgery patients and standardizing routines may reduce variations in the clinical process and thereby improve quality.
After surgery	Patients discharged from different departments with different routines. Discharge letter was not always in hand when the patient left.	All day-surgery patients are discharged from the day-surgery center through new standardized routines.	
		Discharge letter is written and given to the patient before discharge.	

As part of the interventions, a new day-surgery center was designed within the existing premises. All patients met at this center before their elective operation, and all day-surgery patients were discharged from this center without admission to a surgical ward. A computer application was introduced during the project. It provided an overview of referrals, waiting lists, and surgery schedules in all departments. A new position, a capacity coordinator, was created with the mandate to plan and coordinate the surgery program across different departments 6 months ahead. The implementation of the new pathway began when the new day-surgery center was opened in March 2008.

Another project during the study period reduced turnover time between operations by improving logistics and coordination between the facilities for preparation, surgery, and recovery. In addition, the durations of surgical procedures were continuously monitored to get a more realistic picture of the actual time used, thereby improving scheduling of surgery. Furthermore, one operating theatre was designated for emergency cases.

### Study of the interventions

We collected qualitative and quantitative data from the hospital between April 2010 and February 2012. We obtained the number of planned and performed operations and cancellations per month from the hospital’s patient administrative system, and then calculated the monthly CRs. A cancellation was defined as a planned operation that was cancelled within 24 hours of the scheduled time. We considered a decrease in monthly CRs and an increase in the number of performed operations to be an improvement.

To compare CRs before and after the interventions we used the statistical method recommended by Dexter et al., since this method has been shown to be the most robust for this purpose [16]. We calculated the CRs for each month, and transformed the CRs using the Freeman-Tukey double arcsine transformation, and then applied a Student’s *t*-test on the transformed rates using SPSS 18.0 [16].

The numbers of scheduled and performed operations were not normally distributed; thus we analyzed the differences before and after the interventions with the Mann–Whitney *U* test.

The cancellation rate is nonconformance per unit, and we therefore used a u-chart to analyze whether changes in CRs coincided with the interventions and whether improvements were sustained [17].

Cancellations vary by specialty [18]. The number of performed operations increased after the interventions. Thus, an increase in the number of performed operations at a department with low CRs could disproportionately affect the total number of cancellations at the hospital. To assess this effect, we calculated the expected number of cancellations for each department for the time period after the interventions (i.e., the product of pre-intervention CRs and the number of scheduled operations after the interventions). We then calculated the expected cancellation rate at the hospital for the time after the interventions (i.e., the sum of the expected number of cancellations for each department divided by the total number of scheduled operations).

We recorded the volume of elective surgery, emergency surgery, and consultations at the outpatient clinics, because an increase in the volume of one of these activities could affect the volume of the others. The Department of Ophthalmology, which accounts for less than 1% of the total number of emergency cases, was excluded from the count of emergency cases because of incomplete data. CRs from the odontology unit were not available for the entire period, and it was excluded from our study. Moreover, we recorded the number of cancellations per month due to overriding emergency cases.

Changes in the ratio of capacity and demand could influence the cancellation rate [19]. We measured demand as scheduled operations per month and capacity as hours available for surgery per week and the number of full-time equivalents per year for the involved departments. Due to changes in the data system of the hospital, information about full-time equivalents was only available after January 2008.

Utilization of the list of scheduled operations, and in particular list over-runs, can influence CRs [19]. We recorded the proportion of list over-runs for each department. In line with Pandit et al. [19] a list finishing >10% after the scheduled end time was classified as over-running. Data for these calculations were only available for the time after the interventions. Finally, we recorded the number of cancellations per month caused by the hospital not being able to finish the scheduled list.

We interviewed a strategic sample of employees at the hospital (n = 20) to understand how the pathway for elective surgery was redesigned and to identify factors that had contributed to sustained improvements. Interviewees had different professional backgrounds (i.e., physicians, nurses, secretaries, and leaders) and worked in different departments involved in the clinical pathway. The degree of interviewees’ participation in the improvement projects varied. Some had participated in the design of interventions in their project groups and others were not directly engaged in the interventions. The interviews were semi-structured and based on an interview guide that covered the following topics: local problem, setting, context, intended improvement, planning of interventions, implementation of interventions, outcomes, and efforts to sustain outcomes. The interviews were taped, transcribed, and transferred to HyperRESEARCH 2.8.3 software (ResearchWare, 2009) for coding. We developed an initial coding scheme from the themes in the interview guide, and codes were added as the data were analyzed [20]. We interpreted the relationship between the codes to identify distinctive elements of the interventions and factors that influenced success of the improvement process [21].

The protocol of the study was presented to the regional ethical review board, and a formal ethical review was not deemed necessary. The study was approved by the Norwegian Social Science Data Services. All the interviewees participated voluntarily based on informed consent, and could withdraw from the study at any time.

## Results

**Figure 1:**
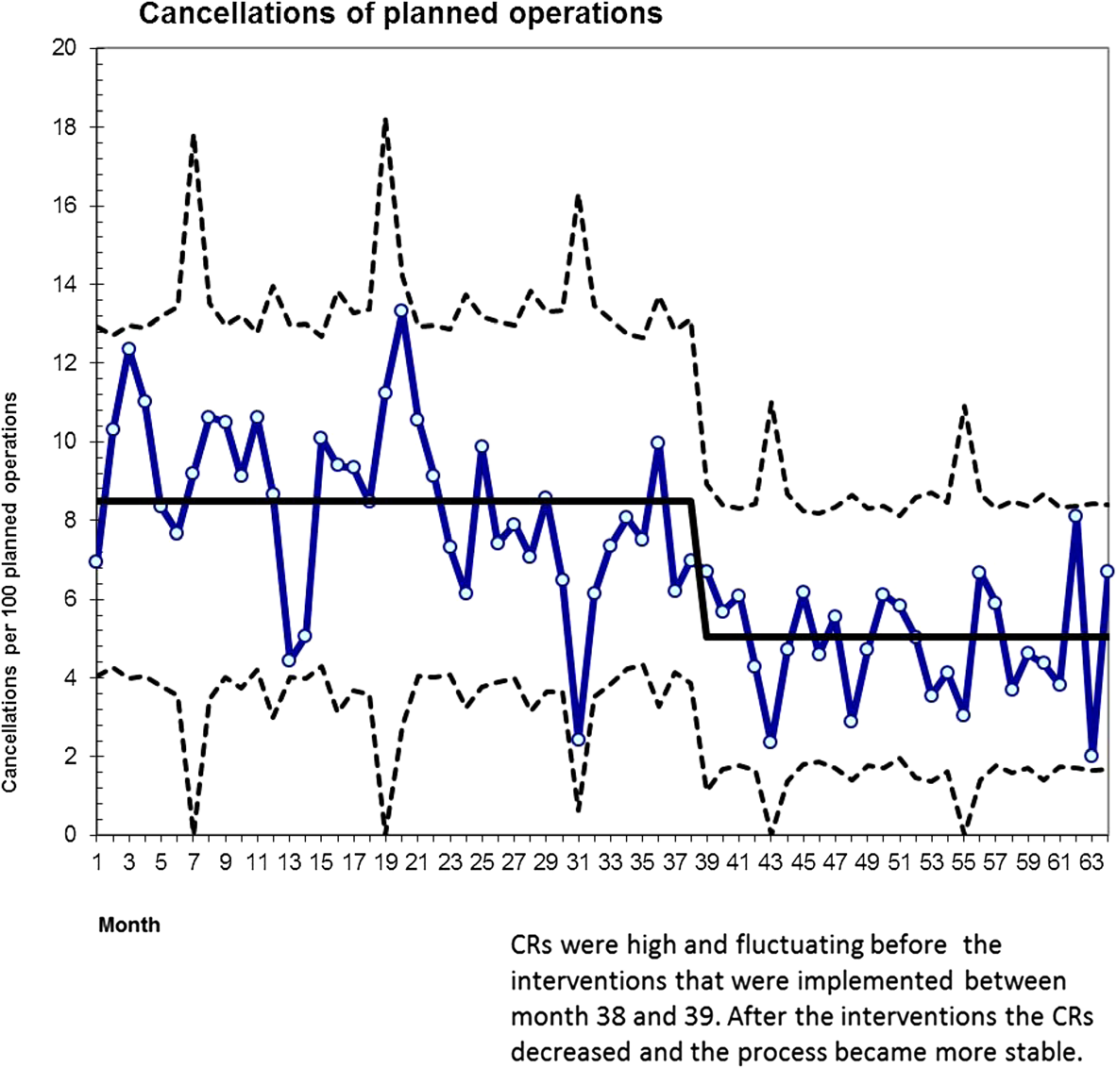
Monthly CRs at the hospital.

The mean cancellation rate was reduced from 8.5% to 4.9% (95% CI for mean reduction 2.6-4.5, *p* < 0.001; Table 3). The u-chart demonstrates a sustained change in CRs that coincided with the interventions at month number 38 (see Figure 1). After the interventions, the CRs were more stable, and all points (registration of monthly CRs) were below the centerline.

**Table 3:** Comparison of outcome measures before and after the interventions

	**Before changes (Jan 2005 – Feb 2008)**	**After changes (Mar 2008 – Apr 2010)**	**P-value (Difference between before and after changes)**
Cancellation rate (mean % per month)	8.5	4.9	<0.001^1^
Transformed cancellation rate^2^	0.30	0.22	<0.001^1^
Total number of scheduled operations (median per month)	373	400	0.04 ^3^
Total number of performed operations (median per month) 323 378 0.04_ 3_	323	378	0.04 ^2^
Emergency cases (median per month) 102 103 1.0 _ 3_	102	103	1.0 ^2^
(Based on time the time period Jan 2006 to Apr 2010)			
Number of consultation at outpatient clinic (mean per month)	2722	3021	0.006 ^1^
(Based on the time period May 2006 to Apr 2010)			

^1^ Student’s *t*-test.

^2^ Using the Freeman-Tukey double arcsine transformation.

^3^ Mann–Whitney *U* test.

The median number of performed operations per month increased by 17%, from 323 to 378, after the interventions (*p* = 0.04; Table 3). The mean number of consultations at the outpatient clinics increased from 2722 to 3021 per month (*p* = 0.006; Table 3). The number of emergency cases per month was the same before and after the interventions (*p* < 0.001; Table 3).

The median number of scheduled operations per month increased from 373 to 400 after the interventions (*p* = 0.04). The capacity increased stepwise until three months after the interventions, from 270 hours per week to 338. At that point it decreased to 304 hours per week and afterwards remained unchanged for the rest of the study period. The number of full-time equivalents as of January 2008 was 279 for the involved departments. For the time period after the interventions the mean number of full-time equivalents was 280 (95% CI 277–283).

Before the interventions the mean number of cancellations caused by the hospital being unable to finish the scheduled surgery lists as planned was 4.2 per month (95% CI 3.1-5.4), and the mean number of total cancellations was 28.1 (95% CI 24.7-31.5). After the interventions the mean number of cancellations caused by the hospital being unable to finish the scheduled surgery lists as planned was 3.1 (95% CI 2.1-4.1, *p* = 0.147). The proportion of over-running lists per department after the interventions was: ophthalmology 1.2%; ear, nose, and throat 2.8%; gynecology 21.1%; general surgery 22.2%; and orthopedics 27.7%.

The mean number of cancellations caused by emergency cases overriding elective surgery was 1.46 (95% CI 0.8-2.1) per month before and 0.1 (CI −0.1-0.4, *p* < 0.001) after the interventions. The expected cancellation rate for the time after the interventions, calculated from pre-intervention cancellation rates and the number of scheduled operations after the interventions, was 8.2%.

Through the analysis of the interviews we found that the following factors were important for the success of the project:

Involvement of frontline professionals in redesigning processes across traditional department borders

Combining professional entrepreneurship with support from staff with knowledge about improvement techniques

Centralizing patient preparation and discharge at one location

Use of computer application to improve planning and coordination of surgery programs across departments

Middle managers role in securing context-sensitive implementation of interventions

Adaptation of interventions based on feedback from frontline clinicians

Before the start of the project, the clinicians agreed that the pathway for elective surgery needed to be improved. However, they had no common understanding of exactly what the problem was or how it could be solved. The top management of the hospital strongly emphasized involving a wide range of frontline professionals from different departments in the project groups. The participants shared information about their everyday work situations and mapped the current state of the pathway by drawing flow charts. The staff from the support unit provided the clinicians with structure and process data from the patient administrative system and guidance about improvement techniques. Through these processes bottlenecks and areas that needed improvement were detected.

All project groups communicated regularly with each other and with the involved departments. Through regular meetings with health personnel affected by the change process, leaders and project groups received feedback on the proposed actions.

The opening of the day-surgery center and the software for surgery planning catalyzed changes in the corresponding clinical processes because participants could no longer follow the old clinical pathway. According to the informants, the degree and speed of change in the clinical processes varied among departments. Despite the involvement of frontline personnel in the planning and decision phases, resistance to change was encountered during the implementation process. Letting patients choose the date for their surgery was especially difficult to implement. The presence of middle managers in the daily work processes allowed them to continuously monitor the degree of implementation and receive feedback on the need to adapt interventions to the local context. Their continued intervention was important to overcome resistance, re-implement changes, and secure context-sensitive implementation and adaptation of changes.

## Discussion

Our study showed a sustained reduction of cancellations over 2 years and an increase in the number of performed operations after the redesign of the surgical pathway. Such a long observation period is rare in research on quality improvement, in which the median follow-up time of the dependent variable is usually less than 1 year [13]. Moreover, the degree of fluctuation of CRs was reduced.

### Causes of the reduction of CRs

The entire pathway for elective surgery, from referral to discharge, was redesigned. Changes were implemented across departmental borders, and frontline staff were broadly involved [22]. Improved management and surgery planning, redesign of work processes, training of staff, and early clinical evaluation have been suggested as strategies to reduce CRs [6]. At our hospital, all of these strategies were applied. The interventions included various elements that were linked to the local context. The distinct effects of the separate elements can therefore not be disentangled.

Sanjay et al. [4] suggest allowing patients to select the time for surgery to give them earlier notice of their operating day, and to send them a reminder. Involving patients in these ways can increase satisfaction with treatment decisions during the initial consultation, which is a strong predictor of whether a patient will attend the surgery [23]. In the new pathway, patients participated in planning the date of their surgery and received the actual date of the operation before they left the outpatient clinic. It is therefore likely that these measures contributed to reducing cancellations.

Early preoperative assessment has previously been shown to reduce CRs [7-10]. Van Klei et al. [8] showed that cancellations attributable to medical reasons decreased from 2.0% to 0.9% for patients who had attended a pre-assessment clinic. Ferschl el al. [7] found a CR of 5.3% among patients who visited an anesthesia preoperative medicine clinic, in contrast to 13.0% for those who did not. Rai and Pandit [9] found that nurse-led pre-assessments in an elective surgical center reduced cancellations. O’Regan et al. [10] demonstrated that a process-oriented multidisciplinary approach for patients who undergo bypass surgery led to improved patient outcomes and lower CRs. Our findings are consistent with these studies.

Cancellations of elective surgery due to emergency cases were practically eliminated after the interventions. The number of emergency cases was the same before and after the interventions and can thus not explain this finding. It is most likely that the designated day-time theatre for emergency cases contributed to reducing CRs, which is supported by previous studies [24,25].

The increase in performed operations exceeded what can be explained by the reduction in CRs alone. More patients were referred to the hospital for elective surgery mainly because of the closure of a local hospital in the region. The number of performed operations increased after the interventions, while the capacity was reduced 3 months after the interventions. This finding indicates that the hospital managed to increase the efficiency of their operating lists [19].

### Alternative explanations for the reduction of CRs

Since the CRs varied across specialties, the disproportional increase in scheduled operations among the surgical specialties could have contributed to reduced CRs after the interventions [18]. The calculated expected CR after the interventions for the hospital was 8.2%, while the observed rate before the interventions was 8.5%. The difference between the calculated and the observed CR indicates that a disproportional increase in scheduled operations may have contributed to the decrease in CRs after the interventions. The effect is, however, small, and can only explain a small portion of the observed reduction in CRs.

A reduction in emergency cases could have affected the CRs. However, the number of emergency cases per month was the same before and after the interventions, making this an unlikely explanation for the reduction in CRs. The increase in the number of performed operations was not caused by a subsequent fall in consultations at the outpatient clinic, as this number increased after the interventions.

Reduced pressure on the service could contribute to reduced CRs [26]. In our case the number of scheduled and performed operations increased. This increase indicates that there was a corresponding increase in demand. The capacity, measured as hours per week available for surgery, increased until 3 months after the interventions and then decreased, while the number of full-time equivalents remained unchanged after the interventions. Thus, the pressure on the service increased after the interventions, making it unlikely that change in the ratio between demand and capacity contributed to reduced cancellations.

An increased tendency to over-run operating lists after the interventions could have contributed to the observed reduction in CRs. Data about this were not available for the pre-intervention period. The proportion of over-running lists for the ophthalmology- and ear, nose, and throat departments was low after the interventions, at 1.2% and 2.8%, respectively. It is therefore unlikely that list over-runs at these departments increased after the interventions, thereby contributing to reduced CRs. For the three remaining departments, we cannot exclude the possibility that list over-runs increased after the interventions. Before the interventions, 4.2 cancellations per month were due to the fact that the hospital was not able to finish the scheduled surgery list as planned. These cancellations could have been avoided by over-running the lists [19]. However, there was no significant reduction of cancellations caused by the hospital not being able to finish the scheduled program after the interventions. This finding indicates that even if list over-runs may have increased after the interventions it cannot be a strong factor for explaining the observed reduction in CRs.

### Factors that contributed to sustained improvements

The redesign of the surgical pathway was embedded in the new strategies to improve the performance of the clinical system. This integration secured a solid foundation in the top management without compromising professional entrepreneurship of middle managers and frontline professionals. Consistent with earlier studies [27,28], we found that this strategy created a basis for improvement by providing guidance about tools and techniques that were important for the success of the project.

The improvement strategy was also important for securing a wide representation of clinical staff in the project groups and for setting the context for the project. In the strategy, the top management emphasized system improvement by equally addressing professional patient and management quality, as well as staff satisfaction. The inclusion of all these dimensions contributed to acceptance by clinicians and other staff, consistent with other studies [12,29].

Improvement groups interacted in an informal network across departments. This network continued after the project period. Frontline employees were engaged in suggesting adoptions and modifications of the interventions. The presence of the middle managers in the actual work processes allowed them to follow the implementation daily and adapt and re-implement changes when needed. The hospital increased the effectiveness of the interventions by adapting them to contextual changes, as indicated by Fixsen et al. [30]. This flexibility seems to be a key factor for sustaining the outcomes.

The optimal use of information technology contributes to the success of high-performing institutions [31]. In our case, the new software for planning surgery integrated the schedules of all the departments thereby improving the scheduling of operations for the whole hospital.

Moreover, the surgery coordinator supported scheduling of operations up to 6 months in advance by matching available slots for surgery and the expected duration of procedures based on previous experiences. The planning processes became more dynamic because waiting lists were taken into account when assigning slots for surgery. Altogether, these measures ensured a better utilization of the total capacity of the operating theatres.

CRs of approximately 5% in our study are still high compared to van Klei et al., [8] who reported a rate below 1%. Further improvements can probably be achieved by fully implementing the aforementioned changes. The process of scheduling surgery could likely be further improved by applying the approach described by Pandit and Tavare [32] using a formula to predict the likely duration of an operating list.

### Limitations

An observational and retrospective study design has the limitation of information bias and confounding, and we cannot prove causality between interventions and the observed outcomes. The improvement project in our case is an example of complex, context-dependent interventions that evolved over time. Such projects are less suitable for strictly controlled, prospective, experimental study designs that could avoid these limitations [33]. Nonetheless, our design makes long-term follow-up feasible and allows us to learn from a successful case by combining qualitative and quantitative data and different analytical methods [34]. Therefore we found it appropriate to use the chosen design.

Our calculation of the number of full-time equivalents before the interventions was based upon data from January 2008, because prior data were not available. These data might not be representative of the pre-intervention period. However, the mean number of full-time equivalents after the interventions was 280 compared with 279 before the interventions, indicating no increase during the post-intervention period. This finding is also supported by data from the interviewees stating that there was no substantial change in the number of full-time equivalents during the study period.

We used the number of scheduled operations per month as a measure for demand. A more precise measure for demand would have been minutes of operating capacity needed per week, but data about this were not available [26]. However, we argue that the number of scheduled patients per month also reflects service demand. Since the number of scheduled patients increased after the interventions, while the capacity was reduced, it is likely that the pressure on the service increased.

In summary, the strength of our study is the long observation time with sustained improvements. Moreover, through our use of quantitative and qualitative methods, we were able to identify factors that contributed to the changes. By using statistical process control we could demonstrate a clear association between interventions and improvements. Data from interviews were consistent across departments and professional borders and did not reveal other organizational changes or quality improvement projects that could have influenced CRs. Finally, our findings are consistent with previous studies. The hospital in our study resembles other district hospitals and the interventions implemented here can likely be adapted to other hospitals of similar size and complexity.

## Conclusion

The redesign of the pathway for elective surgery contributed to a sustained reduction in CRs and an increased number of performed operations. The improvement strategy sought to improve system performance through the involvement of frontline clinicians, use of information technology, and engagement of middle managers, all of which were important factors for the sustained reduction in cancellations of elective surgery.

## Competing interests

The authors declare that they have no competing interests.

## Authors’ contributions

EH had full access to all the data in the study and takes responsibility for the integrity of the data and accuracy of the data analysis. EH, OB, and KH conceived and designed the study. EH acquired the data. EH, CVP, and ABA analyzed and interpreted the data. EH, CVP, ABA, and KH drafted the manuscript. EH, CVP, OB, and KH critically revised the manuscript for important intellectual content. EH and CVP provided statistical expertise. OB and EH obtained funding. CVP, ABA, OB, and KH supervised the study. All authors read and approved the final manuscript.

## Pre-publication history

The pre-publication history for this paper can be accessed here:

http://www.biomedcentral.com/1472-6963/12/154/prepub
